# Prostate cancer with cyst formation detected by whole body positron emission tomography/computed tomography: A case report

**DOI:** 10.3892/ol.2014.2510

**Published:** 2014-09-08

**Authors:** HAKUSHI KIM, SUNAO SHOJI, TETSURO TOMONAGA, MASANORI SHIMA, TOSHIRO TERACHI, TOYOAKI UCHIDA

**Affiliations:** 1 Department of Urology, Tokai University Hachioji Hospital, Hachioji, Tokyo 192-0032, Japan; 2 Department of Urology, Tokai University School of Medicine, Isehara, Kanagawa 259-1193, Japan

**Keywords:** prostate cancer, cyst formation, positron emission tomography/computed tomography

## Abstract

The present study reports a case of prostate adenocarcinoma with cyst formation. A 72-year-old male diagnosed with multiple lung metastases at a local clinic was referred to Tokai University Hachioji Hospital (Tokyo, Japan) for detection of a primary lesion. Whole body positron emission tomography/computed tomography showed strong accumulation of [^18^F]-fluoro-deoxy-2-glucose (FDG) in the small pelvis, and pelvic magnetic resonance imaging revealed a 60×40-mm cystic lesion, with an irregular thickened wall, behind the prostate. The serum prostate-specific antigen (PSA) level was elevated to 211.99 ng/ml, therefore, the patient underwent a transperineal prostate needle biopsy, and was diagnosed with prostate adenocarcinoma with cyst formation. Androgen deprivation therapy was administered for 8 months following the diagnosis of prostate cancer; consequently, the cyst reduced in size and the serum PSA level decreased to 0.14 ng/ml. To the best of our knowledge, this is the first report of a malignant prostatic cyst detected by FDG-positron emission tomography/computed tomography.

## Introduction

Cysts that form in patients with prostate cancer are a type of acquired cyst (1). In prostate cancer, cysts are either secondary cysts caused by intra-cancerous tissue hemorrhage or central necrosis of the cancer tissue, or primary cysts associated with the cancer (2). The majority of cysts that form in prostate cancer patients are secondary cysts (3). In Japan >100 cases have been reported of prostate cancer with cyst formation (3,4). The majority were symptomatic and detected by ultrasound or computed tomography (CT). In total >50% of reported cases presented with metastatic cancer and endocrine therapies were selected for the patient and surgery was only performed for localised disease (5). Nearly all patients were diagnosed with conventional acinar adenocarcinomas from the histology. However, papillary cystadenocarcinomas, embryonal rhabdomyosarcoma and phyllodes tumors are rare (5–7). The present study reports a case of cyst formation in a patient with prostate cancer, secondary to a conventional adenocarcinoma. The patient provided informed consent.

## Case report

**Figure 1:**
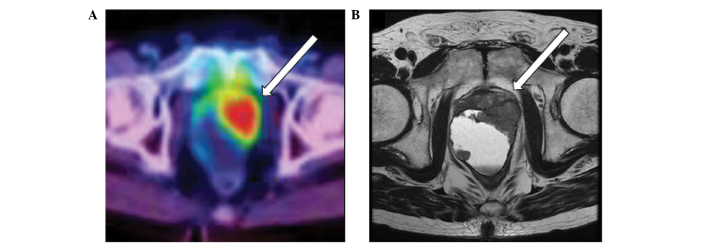
(A) [^18^F]-fluoro-deoxy-2-glucose positron emission tomography/computed tomography showing strong accumulation in the pelvis (arrow), and (B) pelvic magnetic resonance imaging showing a cystic lesion (arrow) behind the prostate gland. The wall of the cyst is partially thickened, and the surface of the thickened wall is irregular.

**Figure 2:**
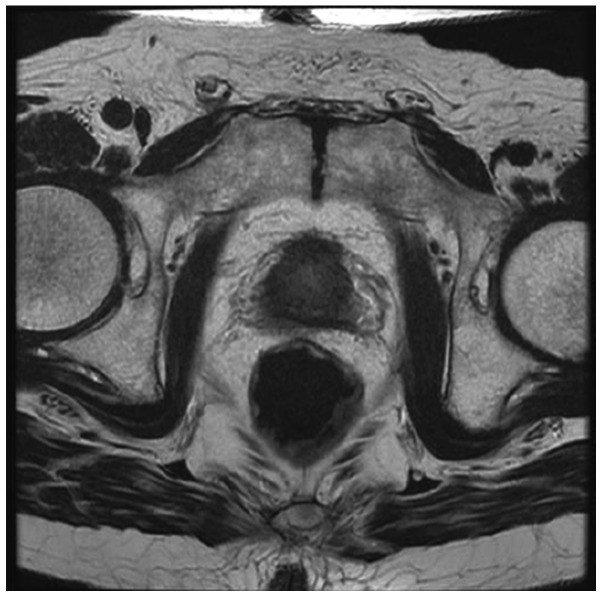
Pelvic magnetic resonance imaging following 8 months of androgen deprivation therapy showing shrinkage of the cyst.

A 72-year-old male was diagnosed with multiple lung metastases by chest radiography and CT during a health examination at a local clinic. The patient was referred to Tokai University Hachioji Hospital (Tokyo, Japan) for examination and diagnosis of the primary tumor. Whole body [^18^F]-fluoro-deoxy-2-glucose positron emission tomography (FDG-PET)/CT showed strong accumulation in the pelvis (Fig. 1). Pelvic magnetic resonance imaging (MRI) revealed a 60×40-mm cystic lesion, with an irregular thickened wall, behind the left lobe of the prostate (Fig. 1); this finding was consistent with the FDG accumulation observed on PET/CT. A transperineal needle biopsy was performed once the serum prostate-specific antigen (PSA) level was found to be elevated to 211.99 ng/ml (normal range, 4.0 ng/ml). Histological examination of the needle biopsy specimens of the cystic wall and prostate gland revealed moderately-differentiated adenocarcinoma (Gleason score 4+3) (8). The contents of the cyst were bloody. The cytological findings revealed no malignancy, but the PSA level of the cystic contents was 45,130 ng/ml. Whole body CT and bone scans revealed no metastasis other than that in the lung, and the patient was diagnosed with prostate cancer with multiple lung metastases. Following 8 months of androgen deprivation therapy (ADT), the cyst shrank (Fig. 2) and the serum PSA level decreased to 0.14 ng/ml. At the 24-month post-ADT follow-up examination, the PSA level was 0.19 ng/ml and the cyst continued to shrink.

## Discussion

In prostate cancer, cysts are considered either secondary cysts caused by intra-cancerous tissue hemorrhage or necrosis of the cancer tissue, or primary cysts associated with cancer (2). In a recent Japanese study of 96 patients with prostate cancer with cyst formation, the majority were symptomatic (dysuria was reported in 56.2% of cases and hematuria in 20.8% of cases), more than half presented with metastatic cancer (54.1%) and almost all were diagnosed with secondary cyst formation (82.1%) (3). In the present case, although a cyst with a diameter of ~6 cm was detected in the small pelvis, the patient was asymptomatic.

It is difficult to predict tissue type without histological examination by MRI or PET/CT. Although only the pathological data acquired by prostate biopsy was obtained, the cystic fluid was bloody with a high PSA level. Based on these results, we speculate that the cyst was a secondary cyst that formed in the prostate cancer patient.

The various histological types of malignant lesions with gross cystic sections in the prostate have been previously reported in the literature, and include conventional acinar adenocarcinomas, papillary cystadenomas (4), embryonal rhabdomyosarcomas (6) and phyllodes tumors (7). Ideally, the histological type of prostate cancer should be determined prior to the start of treatment. The present patient did not exhibit the typical clinical manifestation of conventional acinar adenocarcinoma, but the pathological examination indicated acinar prostate adenocarcinoma. Therefore, the standard therapy for metastatic prostate adenocarcinoma was chosen, which was extremely effective.

FDG-PET/CT is of limited value for detecting prostate cancer, as only ~1% of prostate cancer lesions are FDG-avid (9) and benign conditions of the prostate can also show increased FDG uptake (10). However, there is evidence in the literature that FDG-PET/CT sensitivity and a positive predictive value to detect prostate cancer is increased up to 80 and 87%, respectively, in tumors classified with a Gleason score of ≥7 (11). In the present case, the thickened wall of the cyst that continued to the left lobe of the prostate showed a high accumulation of tracer, which aided in the decision with regard to which part of the body to examine to identify the primary lesion of the lung metastases.

## Abbreviations

FDG: [^18^F]-2-fluoro-2-deoxyglucose
PET: positron emission tomography
CT: computed tomography
MRI: magnetic resonance imaging
PSA: prostate-specific antigen
ADT: androgen deprivation therapy
